# Targeting Transcutaneous Spinal Cord Stimulation Using a Supervised Machine Learning Approach Based on Mechanomyography

**DOI:** 10.3390/s24020634

**Published:** 2024-01-19

**Authors:** Eira Lotta Spieker, Ardit Dvorani, Christina Salchow-Hömmen, Carolin Otto, Klemens Ruprecht, Nikolaus Wenger, Thomas Schauer

**Affiliations:** 1 Department of Neurology, Charité–Universitätsmedizin Berlin, Charitéplatz 1, 10117 Berlin, Germany; eira-lotta.spieker@charite.de (E.L.S.); christina.salchow@charite.de (C.S.-H.); carolin.otto@charite.de (C.O.); klemens.ruprecht@charite.de (K.R.); nikolaus.wenger@charite.de (N.W.); 2 Control Systems Group, Technische Universität Berlin, Einsteinufer 17, 10587 Berlin, Germany; ardit.dvorani@campus.tu-berlin.de; 3 SensorStim Neurotechnology GmbH, c/o TU Berlin, Einsteinufer 17, 10587 Berlin, Germany

**Keywords:** transcutaneous spinal cord stimulation (tSCS), acceleration, mechanomyography (MMG), supervised classification, multiple sclerosis (MS), spinal cord injury (SCI), machine learning

## Abstract

Transcutaneous spinal cord stimulation (tSCS) provides a promising therapy option for individuals with injured spinal cords and multiple sclerosis patients with spasticity and gait deficits. Before the therapy, the examiner determines a suitable electrode position and stimulation current for a controlled application. For that, amplitude characteristics of posterior root muscle (PRM) responses in the electromyography (EMG) of the legs to double pulses are examined. This laborious procedure holds potential for simplification due to time-consuming skin preparation, sensor placement, and required expert knowledge. Here, we investigate mechanomyography (MMG) that employs accelerometers instead of EMGs to assess muscle activity. A supervised machine-learning classification approach was implemented to classify the acceleration data into no activity and muscular/reflex responses, considering the EMG responses as ground truth. The acceleration-based calibration procedure achieved a mean accuracy of up to 87% relative to the classical EMG approach as ground truth on a combined cohort of 11 healthy subjects and 11 patients. Based on this classification, the identified current amplitude for the tSCS therapy was in 85%, comparable to the EMG-based ground truth. In healthy subjects, where both therapy current and position have been identified, 91% of the outcome matched well with the EMG approach. We conclude that MMG has the potential to make the tuning of tSCS feasible in clinical practice and even in home use.

## 1. Introduction

Damage to the upper motor neurons can lead to gait deficits, spasticity, or even full loss of motor function. Transcutaneous spinal cord stimulation (tSCS) constitutes a promising, noninvasive therapy option for these patients. Several studies describe a decrease in spasticity and even enhancement of voluntary movement through tSCS in patients with spinal cord injury [1,2,3,4,5,6]. Moreover, recent investigations have shown similar results in patients with multiple sclerosis (MS) [7,8], reporting a reduction in spasticity as well as an increase in gait velocity and improvement of postural stability.

For tSCS application, an adhesive hydrogel electrode on the lumbar spinal cord and counter electrodes on the abdomen or iliac crest conduct current through the upper body [5]. The objective is to target large- to medium-diameter afferent nerve fibers within the posterior roots, which are known to have enhancing effects on motor control and inhibiting effects on lower limb spasticity [6,9]. Prior to the application of tSCS therapy, a suitable electrode position along the spine and a therapy intensity need to be identified individually for each patient. To prevent discomfort or pain during the therapy in patients with intact body sensation, an electrode location at which a preferably low current activates afferent fibers is desired. Typically, a calibration procedure is conducted using double stimulation pulses of increasing intensity while recording the electromyogram (EMG) of posterior root muscles (PRMs) in the legs. The EMG responses evoked by the first stimulus and their suppression after the second stimulus indicate the recruitment of afferents [10]. The placement of the back electrode can then be adjusted in a manual trial-and-error manner [8] or in an automated process [11]. In the latter, the EMG responses are classified according to the amplitude of the response to the first and suppression characteristics of the response to the second stimulation pulse. The use of EMG sensors involves time-consuming skin preparation with disinfection and abrasive paste, as well as electrode placement for several leg muscles. This procedure is usually required to take place in an expert environment to position the sensors and interpret the signals (in the case of a manual procedure). Thus, current calibration approaches are not suitable for home use and continue to hold great potential for simplification.

In this paper, we introduce a concept for simplification and automation of the calibration procedure. With this novel approach, we strive to simplify the measurement setup by using mechanomyography (MMG) instead of EMGs. Mechanomyography describes the recording of mechanical muscular activity by means of accelerometers, piezoelectric sensors, or condenser microphones [12]. The applications range from fatigue detection [13,14] and examination of neuromuscular disorders [15] to prosthetic control [16]. Furthermore, previous studies have investigated MMG signals in the context of electrical evoked muscle contractions [17,18].

In our approach, we use an accelerometer-based sensor setup for activity recordings of PRMs. The accelerometers are integrated in inertial sensors, also known as inertial measurement units (IMUs) or wearables. We propose a new stimulation procedure that differs from the exclusively EMG-based tuning. In addition to double stimuli, we also apply single-pulse stimulation. We subtract the MMG response of the single stimulus from the response of the double stimulus to extract the acceleration caused by the second pulse of the double stimuli. Such a procedure is necessary because acceleration responses take much longer than EMG responses and therefore overlap in the recordings.

Calibration procedures with healthy individuals and MS patients were conducted while recording EMG and acceleration data from selected PRMs. For the first time, we deployed a machine learning (ML) approach on these data in order to automatically classify the recorded acceleration when considering the EMG classes as ground truth. Not only do IMUs simplify the process, as no skin preparation and electrode placement is necessary, but they are also associated with ecological and economical advantages, as the time savings and simplification during the preparation process decrease demand for expert knowledge and the personnel expenses. Moreover, disposable materials as EMG electrodes are avoided, which decreases the long-term costs and improves sustainability.

## 2. Materials and Methods

The presented approach involved several steps, which we describe extensively in the subsections following this summary. The investigation starts with EMG and IMU sensor data acquisition from the leg muscles during a tSCS calibration procedure on healthy subjects and MS patients. Subsequently, the recorded EMG and acceleration data were passed through a preprocessing pipeline. Here, we cleaned and filtered the data. In addition, the EMG signals were separated into different classes depending on the EMG’s amplitude characteristics. These class labels describe the occurrence or absence of a muscular response after a tSCS stimulus and were considered as ground truth for the subsequent machine learning approach. With this approach, we want to investigate whether muscular responses visible in acceleration signals can be classified in accordance with the EMG-based class labels. For that, we extracted characteristics (features) in the time and frequency domain from all muscular responses after a tSCS stimulus in the acceleration signals. Additionally, the metadata of each participant (e.g., age, height) as well as stimulation properties were added to this feature table. By grouping these extracted features, we generated two different feature sets. After that, three machine learning algorithms were trained and tested using the previously generated feature sets and the ground truth class labels. We trained and tested all algorithms on three datasets: only patient data, only data from healthy subjects, and a mixed dataset consisting of both groups. The output of the different algorithms includes class labels for each recorded acceleration signal. By comparing the ground truth labels and the output, we can estimate the accuracy of each algorithm. During a calibration procedure, the detected response classes provide information on each subject’s individual optimal therapy intensity and electrode position. To evaluate the performance of the classification algorithms in the context of the application, we determined these parameters from the ground truth class labels as well as from the class labels returned by the machine learning algorithms. All steps are subsequently described in more detail.

### 2.1. Data Acquisition Protocol

**Figure 1:**
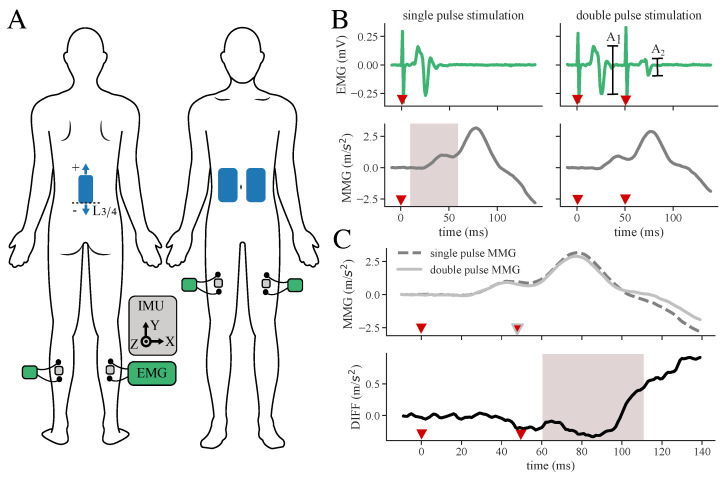
Measurement setup and determination of response suppression from subtracted MMG signals. (**A**) Sensor and electrode placement: Stimulation electrodes (blue) are placed on the back and abdomen. IMUs (gray) are located in between EMG (green/black) electrodes on the selected muscles. (**B**) Filtered and averaged EMG and MMG responses of double as well as single-pulse data. Amplitude characteristics of $A_{1}$ and $A_{2}$ of the double-pulse data are used to classify the EMG signal, while signal characteristics of single and double-pulse data are extracted from the acceleration signals as features for the machine learning algorithm (example signals extracted from the dataset of S2). The gray area indicates time windows for feature extraction. Red triangles mark the stimulation pulses. (**C**) Subtraction DIFF between MMG double and MMG single-pulse muscle response to evade superposition of responses. The gray area indicates time windows for feature extraction. Red triangles mark the stimulation pulses.

A tSCS calibration process was conducted on 11 healthy volunteers (female: 4, male: 7, age: 34.2 ± 6.9 years) and 11 MS patients (female: 6, male: 5, age: 55.9 ± 9.2 years). Individual participant characteristics are shown in Table 1. Each healthy subject underwent several calibration sessions with varying back electrode positions. The patient dataset consists of two data recordings recorded on two different days for each individual patient, to whom only one electrode position was applied per recording day. These data originate from an ongoing study, which examines the immediate effect of tSCS on gait and spasticity in MS patients. As the tuning process is not the primary focus in this study, and we strive to keep the demands of the individual patient at a low level, the number of electrode positions is minimal. However, to still investigate the electrode positions, data from several positions were recorded within the healthy cohort. In order to place a 5 × 10 cm hydrogel stimulation electrode (axion GmbH, Leonberg, Germany) on the spine, the intervertebral space L3/L4 was identified through palpation. After disinfecting the skin, the back electrode was placed on the spine. For the healthy participants, it was placed successively at three different positions: with the lower edge of the electrode 4 cm caudal to L3/L4, on L3/L4, and 4 cm cranial to L3/L4. For the subjects S4 and S10, an additional fourth calibration session with the electrode located 8 cm cranial to L3/L4 was conducted. We chose an additional, even more cranial, position due to lack of reflex activity in the quadriceps muscle, indicating that the three previously recorded positions would not be ideal for a potential therapy application. Regarding the MS patient group, only one electrode position was tested per recording day, varying from 0–4.5 cm cranial to L3/L4. Two interconnected counter electrodes of size 12 × 7 cm (axion GmbH, Leonberg, Germany) were positioned on the abdomen (cf. Figure 1A).

During one calibration session, three double (inter-pulse interval of 50 ms) and three single biphasic pulses (1 ms per phase) were applied with 5 s in between the stimulation incidences with the RehaMove3 stimulator (Hasomed GmbH, Magdeburg, Germany). This stimulation pattern was repeated with increasing stimulation intensities using an increment of 5 mA and starting at an intensity of 5 mA. The examiner terminated the calibration process when reaching the subject’s discomfort level.

**Figure 2:**
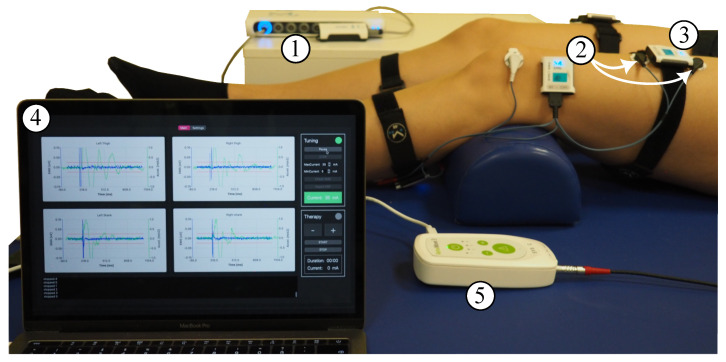
Measurement equipment: MuscleLab synchronization unit (1) and wireless two-channel EMG sensor (2) with one of its channels connected to the quadriceps muscle (white arrows) and the other channel connected to the triceps surae muscle; IMU sensor (3) placed in between the EMG electrodes; laptop with user interface (4) showing the raw signals of acceleration and EMG for all four recorded muscles upon receiving the data after the stimulus; and RehaMove3 stimulator (5) connected to electrodes on the back and abdomen. The stimulator and sensor synchronization unit are connected to the laptop.

Electrical and mechanical muscular responses of the quadriceps (Q), specifically the rectus femoris muscle, and triceps surae (TS) were bilaterally recorded by means of synchronized EMG sensors and IMUs (MuscleLab, Ergotest Innovation AS, Stathelle, Norway). The sample rates were 1 kHz for the EMG and 0.5 kHz for the IMU, respectively. Before the EMG electrode placement, the examiner prepared the skin using disinfection and abrasive paste. The IMUs were placed in between the electrodes of the EMG sensors (Hydrogel Kendall H124SG, Covidien LLC, Mansfield, OH, USA) using elastic straps to ensure that signals of the same muscles were acquired. The complete electrode and sensor setup is shown in Figure 1A. The stimulation and data acquisition during the calibration process were controlled via a customized QT-user interface (QT version 6.3.0 for macOS) programmed in C++11. Figure 2 shows the full measurement and stimulation equipment.

### 2.2. Preprocessing

To classify the EMG responses according to the response amplitude characteristics, the recorded EMG signals during double-pulse stimulation pass through a data processing pipeline implemented in Python 3.9 and adapted from our previously published algorithm for EMG-based analysis [11]. Firstly, the stimulation artifacts are detected in the EMG to synchronize the sensor acquisition precisely with the stimulation. An artifact is marked if the double derivative of the EMG signal is greater than a threshold. Subsequently, the EMG signals are cropped to a common time window, starting 10 ms before the first stimulus and ending 400 ms after the first stimulus. The cropping is followed by a 50 Hz anti-humming [19] and a sliding-median filter with a kernel size of 31 samples. The latter serves as a high-pass filter without applying a ringing effect on the signal. Finally, a similarity check among the three repetitions is conducted with connectivity matrices. Only repetitions with a sufficiently high coefficient of determination between them are averaged. If none of the repetitions are similar, the corresponding signal is marked as faulty. The averaged signals can then be classified according to the characteristics of the peak-to-peak response of the first ($A_{1}$) and the suppression (*S*) of the second pulse ($A_{2}$) [11], with *S*:

(1) $$\begin{array}{cc} S & =\left(1-\frac{A_{2}}{A_{1}}\right)\times 100. \end{array}$$

The EMG responses of each muscle and each applied intensity can subsequently be categorized in a 2-class (distinguishing between response and no response) or 3-class (distinguishing between reflex response, direct muscular response, and no response) manner, as shown in Table 2. Note that • class 1 of the 2-class classification is equivalent to (• class 1 ∩ • class 2) of the 3-class classification. The classes assigned to the EMG responses are considered ground truth for the supervised classification routine. By analyzing the distribution of classes in the four muscles along the applied stimulation intensity and the different electrode positions, a suitable stimulation amplitude and electrode location can be chosen.

Regarding the MMG data, only the acceleration in the z-direction corresponding to the axis orthogonal to the skin is considered for the feature extraction. For preprocessing of the MMG responses of double as well as single pulses, the timestamps of the stimulation artifacts found in the EMG signal are used to crop the signal accordingly to the cropped EMG. Subsequently, the average of the first 10 ms is subtracted from the signal as a non-dynamic gravity filter. The similarity check of the three repetitions is conducted again using connectivity matrices. Analogous repetitions are averaged. As shown in Figure 1B, the mechanical muscular responses in the acceleration signal are much longer than the electrical muscle activity of the EMG, and the responses to the first and second impulse overlap. To separate the responses on the first and second pulse of a double stimulus, we applied the following procedure: the response to the first stimulus is directly measured by applying just a single pulse. We then subtract this response from the response of a double-pulse stimulation. The resulting signal (DIFF) represents the response to the second stimulus of a double pulse and enables direct assessment of the response suppression *S* (cf. Figure 1C). Finally, the averaged single- and double-pulse responses as well as the DIFF signal are used to extract features.

### 2.3. Data Distribution

**Figure 3:**
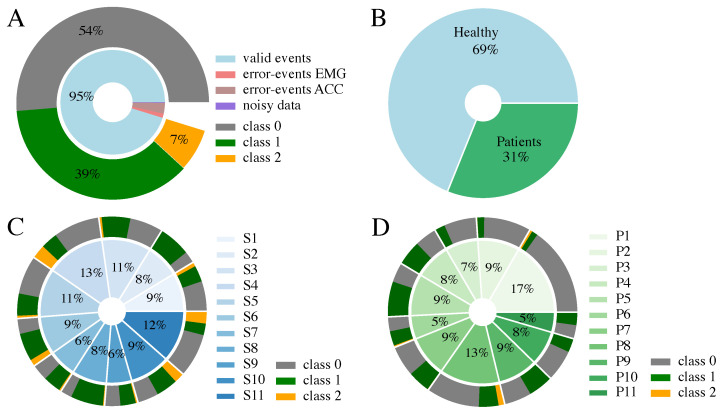
Data composition considering the 3-class EMG classification (Table 2). (**A**) Class distribution of the full dataset; 5% of the data points are invalid due to similarity check errors during averaging or noise in EMG. (**B**) Composition of the 2196 valid events, including proportion of healthy and patient data. (**C**) Proportion each healthy participant contributes. (**D**) Proportion each patient contributes. Please note, that the percentages in (**C**,**D**) are rounded and therefore do not sum up to 100%.

The full dataset used for this classification approach consists of 2196 events. An event comprises an averaged EMG and MMG muscle response to a single-pulse and double-pulse stimulation for a certain muscle at a specific application current and electrode position. The composition of the data is shown in Figure 3. In total, 5% of the data events had to be excluded from the set for the ML-based classification due to errors during the similarity check in the preprocessing pipeline of the EMG and MMG signal, as none of the repetitions were marked as similar. These errors may occur due to movement of the subject during data acquisition, due to occurrence of spontaneous rhythmic muscle twitches, or due to failure of the stimulation artifact detection in the EMG signal. Less than 1% was excluded because of noise in the averaged EMG signal, also resulting from spontaneous muscular activity differing in the three repetitions. The remaining data exhibit an uneven distribution of the ground truth classification. Class 0 is dominant, with 54% of the data belonging to this class, followed by class 1 with 39%, and class 2 with only 7% of all events. As the maximum current applied during the tuning process varies among the participants, the proportion that the individual subjects contributes to the dataset ranges from 6–13% for the healthy participants and 5–17% for the patients.

### 2.4. Feature Extraction

We conceived a list of features in the time and frequency domain to assess mechanical muscle activity characteristics from the MMG. For each evaluated electrode position and stimulation current, these features were extracted for all investigated muscles. Features can be sorted into different categories and serve as input to the supervised classification approaches:

- meta: subject’s metadata, e.g., age, height (four features);
- stim: stimulation parameters: current and electrode position (two features);
- MMG data: sensor data information from MMG (30 features).

The full list of features is displayed in Table 3. The amplitude features, such as MMG single median or MMG DIFF rms, are determined on a window basis from the MMG signals. For the thigh muscles (quadriceps), we chose a window of 10–60 ms after the first stimulation pulse (winA1) for features extracted from the MMG single-pulse response, starting before the onset of the MMG response and ending before the onset of possible muscle response to stimulus two in the double-pulse data. The corresponding window of 10–60 ms after the second stimulus (winDIFF) is selected for amplitude feature extraction from DIFF. For the calf muscles (triceps surae), we shifted both windows, winA1 and winDiff, 5 ms to the right, as the response onset is delayed compared to the thigh muscles. The features 35 and 36 in Table 3 are extracted from the second sensor of the same leg and not from the sensor from which all other MMG data features are determined. These two features were chosen as an approach to perceive possible crosstalk of other muscle activation. To simplify and automate the application of tSCS therapy, we created machine learning approaches using MMG signals and corresponding EMGs recorded from PRMs during double and single tSCS pulses. The EMG responses could easily be sorted into classes based on the amplitudes of first and second pulse responses. These classes were taken as ground truth for the supervised classification algorithms. For the ML-based classification approaches, two combinations of different feature categories were considered. MMG data & meta (SET-OBSERVE), as an observation approach as well as a combination of meta & stim (SET-PREDICT), act as a sensor-less prediction approach without any features extracted from MMG.

### 2.5. ML-Based Classification Approach

We investigated three standard ML approaches to classify the data. The Random Forest (RF) Classifier, the Support Vector Machine (SVM) classifier, as well as Linear Discriminant Analysis (LDA) were considered as possible solutions to the classification problem. The algorithms were applied in a leave-one-subject-out (LOSO) cross-validation loop using the python package scikit-learn (1.3.2). Thus, the dataset of each subject serves as test data for a model trained with the data of the remaining subjects. The balanced accuracy score for imbalanced datasets embedded in the sklearn package was chosen as a measure to validate the classification and avoid overestimating the model performance. To investigate the impact of the different subject groups on the classifiers, three datasets serve as an input for all models: the whole dataset including patients as well as healthy subjects (ALL), only data of healthy subjects (HEALTHY), and only data of patients (PATIENTS). Due to the lack of direct muscle responses (• class 2), especially in the patient dataset (cf. Figure 3D), all machine learning models were implemented as a 3-class classification problem as well as a 2-class classification problem (cf. Table 2).

Hyperparameter optimization for the three classifiers was implemented as a random search with 50 iterations. Table 4 shows the optimized parameters. The hyperparameter cross-validation was again realized in a LOSO manner. However, in the hyperparameter LOSO loop, only the training data, which consist of n − 1 subjects, was considered. The hyperparameters were chosen according to the maximum mean balanced accuracy achieved among the LOSO cross-validation sets. Note that the hyperparameters were solely extracted for the 3-class classification problem. The parameters for the 2-class classification were chosen accordingly. The class weights were set to a balanced state according to the class distribution of the respective dataset for all classifiers.

The classification models and their results for the different datasets (ALL, HEALTHY, PATIENTS), the two feature sets (SET-OBSERVE, SET-PREDICT), the 2-class and 3-class classification, and the three classifiers (SVM, RF, LDA) were documented by means of the python package mlflow (2.8.1). Consequently, 36 different combinations of dataset, feature set, number of classes, and classifier were investigated.

### 2.6. Identification of Stimulation Parameters

**Figure 4:**
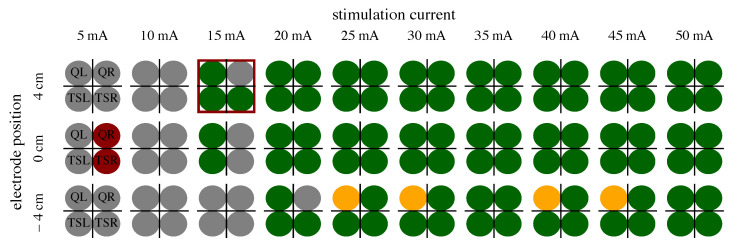
Example of EMG 3-class classification (see Table 2) of S8. The four circles represent the EMG classification determined for the four examined muscles per applied stimulation current and electrode position. The best parameter choice for tSCS is marked with a red frame; the optimal electrode position would be 4 cm cranial to L3/L4, and the optimal therapy current 0.9 × 15 mA.

To find a suitable therapy current and electrode position, the assigned class distribution in the four muscles along the applied stimulation intensity is analyzed for each individual. An example of the class distribution extracted from EMG data with the proposed position and therapy current is presented in Figure 4. For extracting the parameter pair (position, current), the following nested search is applied to each individual subject [11]:

1. At least two • class 1 labels in one current among all muscles present in the respective electrode position;
2. Largest number of class 1 labels per current;
3. Smallest current difference between onset of leg response and presence of maximum number of class 1 labels;
4. Lowest stimulation current;
5. Largest sum of all class 1 labels in the respective position.

We chose a sub-motor-threshold therapy current of 90% of the intensity, where the first reflex occurred [8,11]. By extracting the therapy parameters for both EMG and SET-OBSERVE and SET-PREDICT classifications, the ML-based classification approach and its accuracy can be assessed in the context of tSCS application. Note that, for the patients, only the stimulation current is determined, as only one electrode position is available in the datasets.

## 3. Results

### 3.1. ML-Based Classification Approach

**Figure 5:**
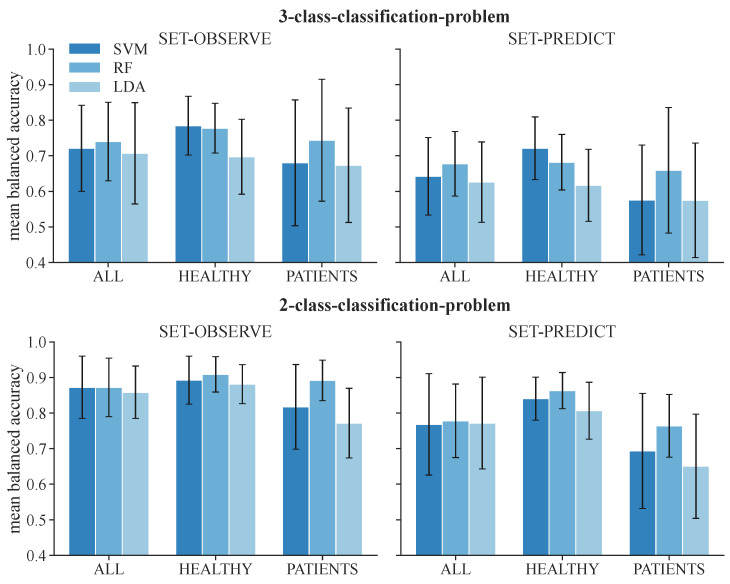
Mean balanced accuracy and standard deviation of test data among all subjects for the 3-class classification and 2-class classification problems.

**Figure 6:**
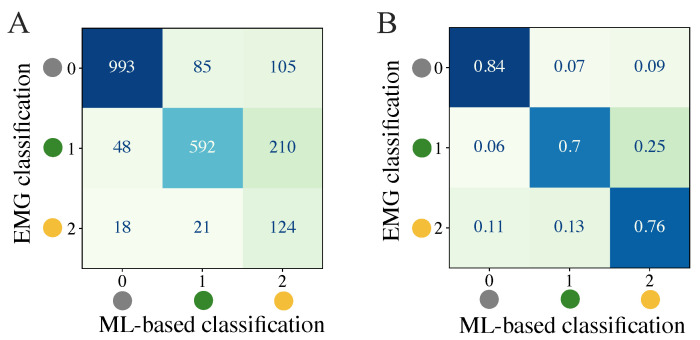
Quantification of classification results through a confusion matrix example for 3-class classification of dataset ALL, feature set SET-OBSERVE, and RF model. (**A**) Summed confusion matrix for all subjects. (**B**) Ratios for each row (ground truth class) among all subjects.

We first attempted to quantify the balanced accuracy of the ML-based classification approaches to determine differences in the results evolved from the different classifiers and datasets. The mean balanced accuracies for the test data of the 3-class classification problem among all subjects represented in the datasets ALL, HEALTHY, and PATIENT are displayed in Table 5. The corresponding results for the 2-class classification problem are shown in Table 6. The mean balanced accuracy and standard deviations are additionally visualized in Figure 5. The feature set that includes acceleration data (SET-OBSERVE) performs better in terms of mean balanced accuracy than the dataset consisting of stimulation information and metadata (SET-PREDICT). A clear performance lead of one classifier type is not visible. For the dataset ALL and 3-class classification, SET-OBSERVE reaches a maximum mean balanced accuracy of 0.74 for the RF, while the maximum value for SET-PREDICT is 0.68, also using the RF classifier. The standard deviation of the PATIENTS dataset is conspicuously higher than for the other datasets. Regarding the 2-class classification, the RF classifier yields the best balanced accuracy for most dataset and feature set combinations. The corresponding maximum mean balanced accuracies are 0.87 (SET-OBSERVE) for the RF and SVM classifier and 0.78 (SET-PREDICT) for the RF classifier. Therefore, we can see a clear increase in performance, differentiating only between response and no response, without distinguishing further between reflex and direct muscular response. This is reflected in an increased mean balanced accuracy as well as a decrease in the standard deviation. The inaccuracies between class 1 and class 2 response classification are also visible in the confusion matrices presented in Figure 6. The example shown refers to results regarding dataset ALL, feature set SET-OBSERVE, and the Random Forest 3-class classifier. The sensitivity of class 0 is higher than for classes 1 and 2 (Figure 6B). In particular, the proportion of falsely classified class 1 events sorted into class 2 is conspicuous. Subsequently, we determine how the reported accuracies affect the identified stimulation parameter.

### 3.2. Extraction of Stimulation Parameters

**Figure 7:**
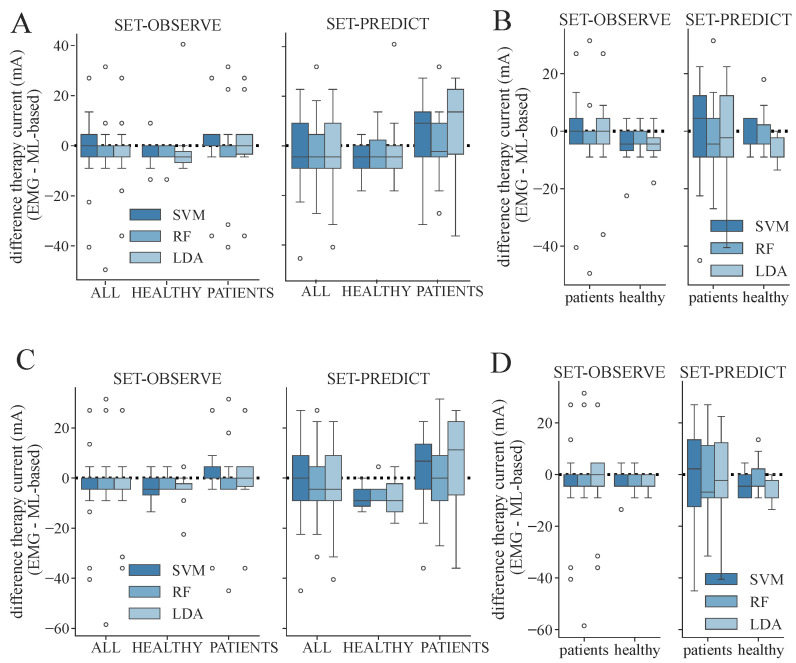
Differences between therapy currents extracted from EMG- and ML-based class distribution for both feature sets and all determined datasets and models. A negative value indicates that a higher therapy current was identified with the corresponding machine learning models compared to using the EMG. The whisker length is set to a maximum of 1.5 × the box height. (**A**) Differences in therapy current (EMG- minus ML-based) for SET-OBSERVE and SET-PREDICT and all datasets regarding the 3-class classification. (**B**) Differences in therapy current for dataset ALL, distinguishing between patients and healthy subjects regarding the 3-class classification. (**C**) Differences in therapy current and all datasets regarding the 2-class classification. (**D**) Differences in therapy current for dataset ALL, distinguishing between patients and healthy subjects regarding the 2-class classification.

**Figure 8:**
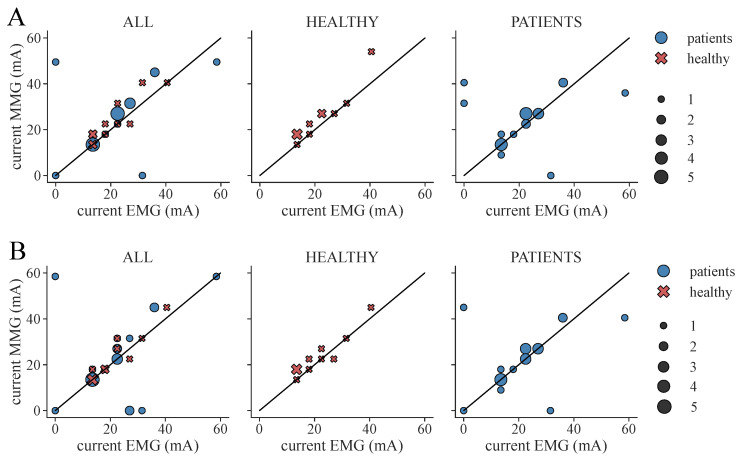
Extracted therapy current from ground truth (EMG) classification and ML-based prediction of feature set SET-OBSERVE for the RF classifier. The black diagonal represents the optimal scatter location, when EMG current and ML-based current are equal. A stimulation current of 0 mA indicates that no suitable stimulation current could be found due to a lack of class 1 classifications. The scatter sizes represent the number of equal samples. (**A**) Therapy currents found for 3-class classification. (**B**) Therapy currents found for 2-class classification.

**Figure 9:**
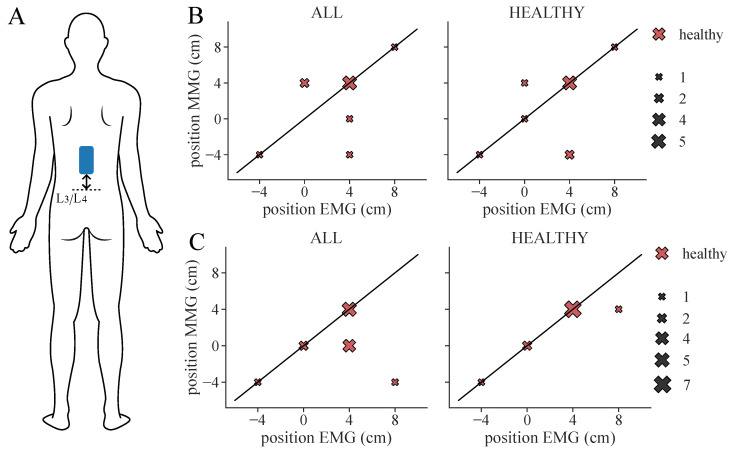
Extracted therapy electrode position from ground truth (EMG) classification and ML-based prediction of feature set SET-OBSERVE for the RF classifier considering all healthy subjects. The black diagonal represents the optimal scatter location, when EMG position and ML-based position are equal. The scatter sizes represent the number of equal samples. (**A**) Electrode location: Position is measured as a distance from L3/L4. (**B**) Therapy position found for 3-class classification. (**C**) Therapy position found for 2-class classification. Note that, for two subjects (S6, S7), two equally suitable locations were found in the ML-based classification, whereas only one suitable position was selected in the ground truth classification.

**Figure A1:**
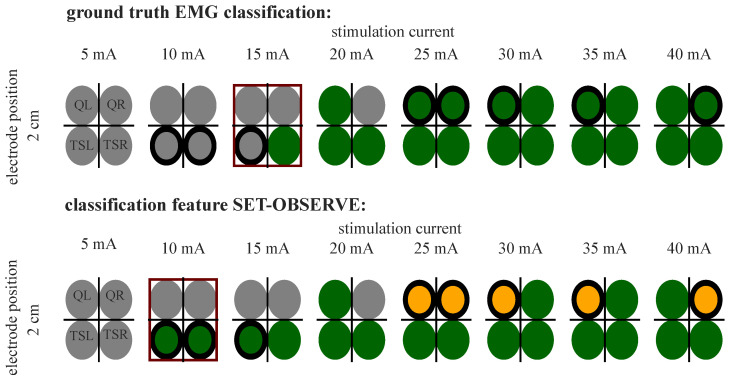
Example of EMG 3-class classification and prediction (SET-OBSERVE, dataset ALL, SVM) (see Table 2) for P5. The best parameter choice for tSCS is marked with a red frame for both classifications. Differences in classification are marked with a bold black frame around the circles.

From the determined ML-based classifications, we identified the suitable personalized therapy current and, in the case of healthy subjects, optimal electrode position, to assess the classifier performances through their application. We extracted suitable therapy currents from the ground truth classification of the EMG as well as from all ML-based classification results for all subjects and measurement days (two for each patient, one for each healthy subject). An example of the classification overview and current extraction is shown in Figure A1. Additionally, the optimal electrode position was determined for each healthy subject. Figure 7 illustrates the difference between the ground truth therapy current and the currents extracted from all ML-based classification results. If no current was found due to a lack of class 1 events, the stimulation current was set to 0 mA. Overall, SET-OBSERVE is superior compared to SET-PREDICT in terms of discrepancy between EMG- and ML-based therapy current. Furthermore, the therapy current for healthy subjects is more accurate compared to the current identified for the patients, which has a higher variance. 2-class and 3-class classifications show a similar parameter distribution. A direct comparison between ground truth and ML-based therapy parameters for the RF classifier is additionally visualized in Figure 8 and Figure 9. Only results of the better performing SET-OBSERVE, which includes MMG data characteristics, are displayed. Most of the extracted currents are located around the optimal diagonal (cf. Figure 8). However, for the patient data, some outliers are located on the x-axis, when no current could be extracted in the ground truth, or y-axis, when no current could be extracted in the ML-based prediction. All outliers on the x-axis originate from one of the measurements of patients P1 and P2, where no stimulation current could be found in the EMG classification. The extracted electrode positions from the classification of SET-OBSERVE are, for the most part, located on the optimal diagonal (cf. Figure 9).

**Figure 10:**
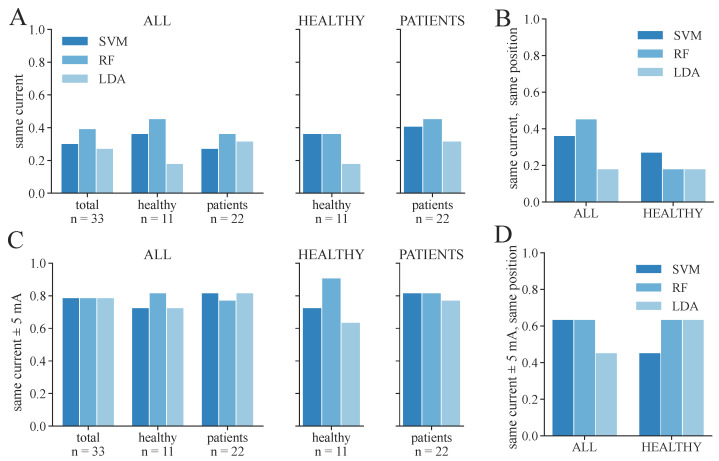
Proportion of correctly identified stimulation parameters from ML-based classification regarding the 3-class classification, considering the parameters extracted from the EMG as ground truth. Only the feature set SET-OBSERVE is shown. (**A**) Proportion of measurements in which exactly the same therapy current was extracted as in the EMG. (**B**) Proportion of measurements in which exactly the same current and electrode position were found as in the EMG; only the proportion of healthy subjects is shown. (**C**) Proportion of measurements in which the found therapy current for ML-based classification was within the current extracted from the EMG ± 5 mA. (**D**) Proportion of measurements in which the found therapy current for ML-based classification was within the current extracted from the EMG ± 5 mA and in which the same electrode position was found as in the EMG; only the proportion of healthy subjects is shown.

**Figure 11:**
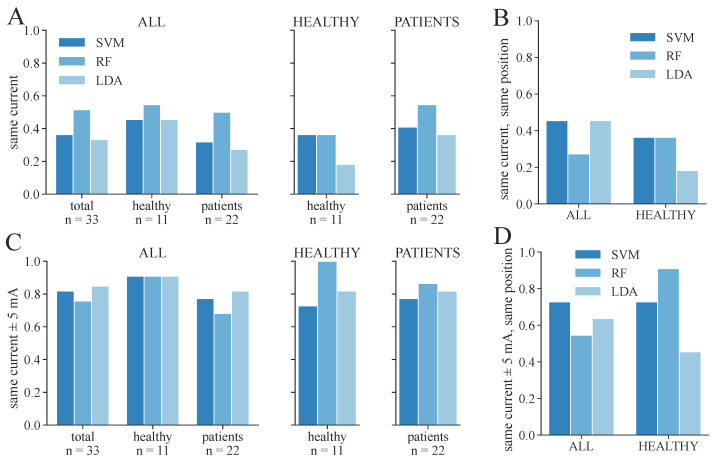
Proportion of correctly identified stimulation parameters from ML-based classification regarding the 2-class classification, considering the parameters extracted from the EMG as ground truth. Only the feature set SET-OBSERVE is shown. (**A**) Proportion of measurements in which exactly the same therapy current was extracted as in the EMG. (**B**) Proportion of measurements in which exactly the same current as well as electrode position was found as in the EMG; only the proportion of healthy subjects is shown. (**C**) Proportion of measurements in which the found therapy current for ML-based classification was within the current extracted from the EMG ± 5 mA. (**D**) Proportion of measurements in which the found therapy current for ML-based classification was within the current extracted from the EMG ± 5 mA and in which the same electrode position was found as in the EMG; only the proportion of healthy subjects is shown.

We additionally conducted a statistical analysis of the proportion of correctly determined therapy parameters for 3-class and 2-class classifications, respectively. These results are shown in Figure 10 and Figure 11. The same therapy current could only be found for a maximum of 36–45% of the subjects/measurement day regarding 3-class classification (Figure 10A), depending on the model and dataset. The highest proportion was achieved in the PATIENTS dataset and the RF classifiers, as well as among the healthy subgroup in the ALL dataset with the RF classifier. The corresponding maximum proportions for the 2-class classifications are 36–59% (Figure 11A). Again, the highest proportion was achieved in the PATIENTS dataset and RF, as well as the healthy subgroup in the ALL dataset. However, these numbers increased to maximum values of 79–91% for the 3-class (Figure 10C) and 82–100% for the 2-class classification (Figure 11C) when considering a small margin of 5 mA around the ground truth current. In both cases, the HEALTHY dataset with the RF classifier yields the best results. For the combined ALL dataset, up to 85% (LDA) of the extracted currents were within the applied margin. The right combination of therapy current and electrode position was determined for up to 45% of the healthy subjects for the dataset ALL and RF with regard to 3-class classification (Figure 10B). The same maximum value was achieved for dataset ALL and the SVM and LDA classifier regarding the 2-class classification (Figure 11B). With the 5 mA margin, these numbers improved to 64% (Figure 10D) and 91% (Figure 11D), respectively. The RF classifiers provide the highest proportion of correct therapy parameters for most datasets regardless of 2- or 3-class classification.

## 4. Discussion

To simplify and automate the application of tSCS therapy, we deployed machine learning approaches to classify a sensor-based feature set containing MMG data characteristics from PRMs to double and single tSCS pulses using the corresponding EMG classes as ground truth. Additionally, a feature set without any sensor information containing only metadata and stimulation information was determined and classified. Through the ML-based class distributions, we identified suitable therapy parameters for each subject and compared these with the ground truth parameters extracted from the EMG classes.

The results show better classification accuracy for the feature set SET-OBSERVE, which includes MMG data, implying the importance of sensory observations to target individual therapy parameters. However, the classifiers for the 3-class classification performed only moderately well for the separation between class 1 (•, reflex response) and class 2 (•, direct muscular response). Thus, the similarity between these classes regarding the MMG in the 3-class classification problem is very high, making a separation challenging. We suspect two reasons for this, as follows.

Firstly, the suppression (1) that is important for the 3-class EMG classification cannot be determined directly from MMG due to the non-linear summation of the forces and, therefore, the accelerations, in the case of sequential induced muscle twitches. With the same muscle activation, and accordingly the same EMG response to the first and second impulse (no suppression), the increase in force or acceleration due to the second impulse can be greater than the response to the first impulse [20,21]. This means that the amplitude of the acceleration in the DIFF signal would be increased compared to the response of the first impulse, despite the same EMG activity. Another problem is that these non-linear effects are strongly dependent on the muscle fiber type [22], and the composition of muscle fiber types can vary among patients. To reduce these non-linear force summations after the sequential muscle twitches, we could apply longer inter-pulse intervals between the double stimulation pulses. However, this would directly influence the suppression effect in the EMG signals, especially for patients with SCI [10]. As we need the post-activation suppression information to distinguish between reflex response and direct muscular response in the ground truth data, lengthening the inter-pulse interval would not be beneficial for therapy parameter identification.

The second reason for inaccuracies when distinguishing between reflex response and muscular response could be crosstalk in the acceleration signals due to movement of joints or contraction of other leg muscles. To provide knowledge on the activity of the other recorded leg muscle to the algorithm, we added two amplitude features of these muscles to the feature set SET-OBSERVE. This was done as a simple attempt to internally track and catch crosstalk. As we fastened the IMUs with elastic straps over the muscle belly, contractions from muscles with direct contact to the strap could disturb the sensor signals further. However, lower limb muscles that were not recorded during the calibration process are part of the PRMs, such as the hamstring or tibialis anterior muscle [23]. Hence, activity in these muscles can still indicate activation of afferent nerves accountable for spasticity reduction and therefore lead to suitable therapy parameters. Nonetheless, as these muscles were not part of our datasets, this assumption is not verifiable and would have to be examined in further investigations. Crosstalk of joint movement resulting from the muscle contractions due to the tSCS pulses is also a possible signal disturbance. There is a considerable delay between a contraction of a distant muscle and a perceptible movement at the sensor. Keeping the measurement interval close to the stimuli should guarantee that only local contraction forces and corresponding accelerations are sensed. If crosstalk is present, then crosstalk from antagonistic muscles across the strap is more likely than from muscles further away, which can only act on the sensor via joint or leg movements with a greater time delay. To avoid big influences of superposition because of different acceleration sources, we chose the windows winA1 and winDIFF to be very close to the pulse artifact and, therefore, to the reflex or muscle activation time stamp.

After the ML-based classification, we further identified individual therapy parameters for each subject from the classification results. The therapy current extracted from ground truth and from ML-based classification corresponded in only a fraction of the subjects. However, most of the determined currents varied around the optimal value. These fluctuations between ground truth and predicted therapy current result from inaccuracies of the classifiers, especially around the applied pulse amplitude at which the first muscle reactions occur. These inaccuracies also transfer to the choice of the electrode position in healthy subjects. However, a position that was not labeled as optimal through the ground truth classification can still be potentially suitable for tSCS when an adapted higher current is applied. Nonetheless, we strive for the most tolerable stimulation for the subject, which we assume can be extracted from the EMG data acquired at different electrode positions. Overall, the presented algorithms show good results for both therapy current and position identification. In order to successfully use MMG for personalized tSCS tuning, synchronized stimulation and IMU recording would be required, as no artifacts are visible in the acceleration signals.

In this paper, we investigated both 3-class classification as well as a simplified 2-class classification. The latter generally produced higher balanced accuracy values and more accurate stimulation parameters. Thus, we pose the question of whether a 2-class classification would be precise enough to activate afferents and, hence, achieve a therapeutic effect after tSCS therapy. Referring to the data we collected in this and in previous investigations, we observe that if a rather big electrode of 5 × 10 cm is placed over the upper lumbar spine, class 1 (reflex) responses will be most probably elicited before class 2 (direct muscular) responses occur. This corresponds with the recruitment order of different nerve fiber types. Large-diameter proprioceptive sensory nerves have the lowest activation threshold and are therefore recruited at lower stimulation intensities than motor fibers [24,25]. To conclude, since we strive for therapy currents at sub-motor level and since the first responses visible during the calibration procedures are most likely reflex responses elicited by afferents, a 2-class classification would presumably be sufficient. However, whether that is true for all groups of patients that would profit from tSCS cannot be confirmed and would have to be determined in further investigations.

The newly introduced method with single- and double-pulse stimulation was intended to improve the classification of muscle and reflex responses in the MMG. However, a disadvantage of this approach is the double time compared to the EMG-based approach, which only includes double stimuli. Given the good performance of tSCS-tuning based on 2-class classification, the question arises whether the inclusion of single-pulse data, i.e., the calculated differences and the corresponding features, is necessary to sufficiently detect any stimulus-induced activity in the MMG data. This open question will be pursued in the future.

For our investigations, we included patient tuning data as well as data from healthy subjects. Due to the limited quantity of our patient dataset, a machine learning approach using exclusively the available patient data would have been unsatisfying. Therefore, we included the larger healthy dataset in order to be able to investigate the performance of our method with several electrode positions. However, our results indicate that the accuracy of the therapy parameter choice is slightly higher for the patient cohort if we train the model solely on the PATIENT dataset instead of on the mixed ALL dataset (cf. Figure 10A and Figure 11A). This could imply that training an individual model for patients with a common medical condition is beneficial. Another patient group that was not investigated in the paper but would profit from a simplified tSCS tuning process is the group of patients with spinal cord injuries. Hence, this cohort should be included in future studies.

Another disparity in the results of the different subject groups is the larger standard deviation of the balanced accuracy among the MS patient group (cf. Figure 5, dataset PATIENTS) compared to the mixed dataset (ALL) and healthy dataset (HEALTHY). A reason for that could be the difference in composition of the two datasets (cf. Figure 3). The total number of events for the patients accounts for only one-third of the overall dataset. Additionally, the proportions of class 1 and class 2 events are smaller in the patient data. Therefore, the transition phase from no response (class 0) to response (class 1 and class 2) during the individual tuning of a patient takes up a larger proportion of the PATIENT dataset. However, in this transition phase, more natural inaccuracies in the classification approaches occur due to the small amplitudes of the responses. The smaller quantity of events and higher proportion of the transition phase could explain the larger standard deviation of the balanced accuracies. Another reason could be the disparity in severity and location of neurological defects among the MS patients, which causes a limited comparability between them. Thus, the LOSO cross-validation performance can vary considerably within the patient group. To conclude, to further improve the performance and reliability of our models, larger datasets from MS patients would be beneficial. Additional models for other patient groups, such as SCI, can be trained when the corresponding data are available.

For MMG, two sensor technologies are commonly used: piezoelectric contact (PEC) and accelerometer (ACC). In [26], the frequency responses of PEC and ACC have been experimentally determined. Results for the sensors without housing restrictions show that the PEC transducer resembled that of the double integral over time of the ACC transducer signal, acting as a displacement meter of muscle vibration. As it correlates better with muscle force, we selected ACC as the sensor technology. However, study [26] indicates the negative influence of housing restrictions on the measurements. We used a medically certified system with synchronous EMG and acceleration recording, as this was mandatory for our investigation. The inertial sensors with accelerometer have a dimension of 41 × 47 mm and a mass of 20 g. These physical quantities result from the use of rechargeable batteries for enabling wireless data transmission. The latter increases the usability of the system. Latencies in data transmission are not critical, as data from the sensors are transmitted with synchronized time stamps batch-wise to the laptop after each applied single or double pulse. Upon receiving the data, non-causal data processing is performed. The sensor size and mass (inertia) might negatively impact the detection of muscle responses as they dampen vibration. Possible advantages of smaller and lighter sensors and different mounting approaches need to be investigated in the future.

As we only looked at a small number of classifiers, there might be other algorithms that reach a similar or even better performance. We chose classifiers that are suitable for multi-class classification and tried to include linear (LDA) as well as non-linear algorithms (RF and SVM with radial basis function kernel) that vary in their computational complexity. Despite their differences, the performance of the chosen classifiers is similar. Hence, we expect that other classifier types would perform comparably. With the choice of our classifiers and their performances, we conclude that machine learning-based classification algorithms are generally suitable for this application.

Recent publications have determined that selective tSCS targets specific muscle groups for patients with SCI [27,28]. In this application, finding the accurate electrode position is more essential than in the calibration procedure that we proposed in this paper, as smaller electrodes or electrode arrays are used to selectively activate muscles. Hence, applying the proposed algorithm to these datasets would be another opportunity to investigate its performance in the context of a more advanced application.

## 5. Conclusions

In this paper, we investigated the suitability of MMG for tuning individual tSCS therapy parameters. Three different classifier types were examined for supervised classification, considering the typically used EMG as ground truth data. We compared the accuracy of the classification for patient data, data from healthy subjects, and for a combined dataset of n = 22. Furthermore, the classifiers were also tested without using any MMG sensor data to observe the performance of the prediction model.

Overall, the classifiers performed better with MMG sensor data present in the training data. The extraction of individual therapy parameters from the classified signals was possible. Both determined therapy currents (for healthy subjects and patients) as well as positions (for healthy subjects) show good results for most of the subjects and models. The algorithm should be further investigated with additional datasets and in different application contexts. We conclude that the presented use of inertial sensors has the potential to tune the tSCS current and position beyond clinical research in clinical practice and even in home use, where EMG-based measurements may be too difficult, time-consuming, or wasteful in terms of required materials.

## Figures and Tables

**Table 1:** Participants; PPMS: primary progressive MS, SPMS: secondary progressive MS.

Subject	Age (Years)	Sex	BMI	Height (cm)	Diagnosis	Dataset
S1	35	M	24.1	173	healthy	3 electrode positions
S2	30	M	21.5	200	healthy	3 electrode positions
S3	38	M	22.5	180	healthy	3 electrode positions
S4	33	M	25.5	175	healthy	4 electrode positions
S5	38	M	24.8	184	healthy	3 electrode positions
S6	33	F	20.7	177	healthy	3 electrode positions
S7	27	F	18.6	159	healthy	3 electrode positions
S8	28	F	20.3	160	healthy	3 electrode positions
S9	25	F	20	173	healthy	3 electrode positions
S10	40	M	21.6	190	healthy	4 electrode positions
S11	49	M	30.7	176	healthy	3 electrode positions
P1	65	M	28.09	176	PPMS	2 datasets of 2 days, 1 electrode position
P2	48	F	25.71	159	SPMS	2 datasets of 2 days, 1 electrode position
P3	66	F	25.76	174	PPMS	2 datasets of 2 days, 1 electrode position
P4	50	F	16.7	173	PPMS	2 datasets of 2 days, 1 electrode position
P5	53	F	19.53	160	PPMS	2 datasets of 2 days, 1 electrode position
P6	35	F	19.5	181	PPMS	2 datasets of 2 days, 1 electrode position
P7	60	M	23.88	165	PPMS	2 datasets of 2 days, 1 electrode position
P8	58	M	20.15	174	PPMS	2 datasets of 2 days, 1 electrode position
P9	55	M	26.01	186	PPMS	2 datasets of 2 days, 1 electrode position
P10	65	M	23.67	178	PPMS	2 datasets of 2 days, 1 electrode position
P11	60	F	19.33	164	PPMS	2 datasets of 2 days, 1 electrode position

**Table 2:** 2- and 3-class approaches, EMG (ground truth) class classification characteristics.

EMG Classification (Ground Truth)			
2-class Classification		3-class Classification	
• class 0:	no response, $A_{1}$ < 50 μV	• class 0:	no response, $A_{1}$ < 50 μV
• class 1:	response, $A_{1}$ > 50 μV	• class 1:	reflex response, ($A_{1}$ > 50 μV) & (S > 60%)
		• class 2:	muscle response, ($A_{1}$ > 50 μV) & (S < 60%)
• invalid data: discarded due to similarity check error in EMG or MMG signal or noisy EMG data			

**Table 3:** Features divided into the categories meta, stim, and MMG data.

No.	Feature Name	Category	Description
1	BMI	meta	body mass index 2
2	sex	meta	0: male, 1: female
3	age	meta	age
4	height	meta	body height
5	position	stim	value in cm distance of lower electrode edge from L3/4
6	current	stim	stimulation current
7	sensor	MMG data	0 for Q; 1 for TS
8	MMG single mean	MMG data	mean in single-pulse signal in winA1
9	MMG single median	MMG data	median in single-pulse signal in winA1
10	MMG single std	MMG data	std in single-pulse signal in winA1
11	MMG single RMS	MMG data	RMS in single-pulse signal in winA1
12	MMG DIFF mean	MMG data	mean in DIFF signal in winDIFF
13	MMG DIFF median	MMG data	median in DIFF signal in winDIFF
14	MMG DIFF std	MMG data	std in DIFF signal in winDIFF
15	MMG DIFF rms	MMG data	RMS in DIFF signal in winDIFF
16	freshnet distance MMG	MMG data	freshnet distance between double- and single-pulse signal in winDIFF
17	p2p MMG single	MMG data	peak-to-peak amplitude in single-pulse signal in winA1
18	p2p MMG DIFF	MMG data	peak-to-peak amplitude in DIFF in winDIFF
19	r^2^ MMG single double	MMG data	squared correlation coefficient between single- and double-pulse data in a window of 50 ms:end
20	r MMG single double	MMG data	correlation coefficient between single- and double-pulse data in a window of 50 ms:end winDIFF
21	mpf double	MMG data	mean power frequency of double-pulse signal
22	mpf single	MMG data	mean power frequency of single-pulse signal
23	mpf DIFF	MMG data	mpf DIFF
24	auc spectral density single	MMG data	area under spectral density curve of single-pulse signal
25	auc spectral density double	MMG data	area under spectral density curve of double-pulse signal
26	auc spectral density DIFF	MMG data	area under spectral density curve of DIFF
27	max spectral density single	MMG data	max in spectral density curve of single-pulse signal
28	max spectral density double	MMG data	max in spectral density curve of double-pulse signal
29	max spectral density DIFF	MMG data	max in spectral density curve of DIFF
30	zcr single	MMG data	zero-crossing rate in single-pulse data
31	zcr double	MMG data	zero-crossing rate in double-pulse data
32	zcr DIFF	MMG data	zero-crossing rate in DIFF
33	max slope DIFF	MMG data	max slope in DIFF in winDIFF
34	max slope single	MMG data	max slope in single-pulse signal in winA1
35	p2p MMG single 2nd muscle	MMG data	peak-to-peak amplitude in single-pulse signal in winA1 of other leg muscle
36	p2p MMG diff 2nd muscle	MMG data	peak-to-peak amplitude in DIFF in winDIFF of other leg muscle

**Table 4:** Choice of hyperparameters for the python classifiers of sklearn.

Model	Python Classifier	Hyperparameters
SVM	SVC	gamma, C
RF	RandomForestClassifier	min_samples_split, min_samples_leave, max_depth, max_samples
LDA	LinearDiscriminantAnalysis	n_components, solver, tol

**Table 5:** Mean and standard deviation of balanced accuracy in test data across all subjects in the corresponding datasets for the 3-class classification approach.

Dataset	Feature Set	SVM	RF	LDA
ALL	SET-OBSERVE	0.72 ± 0.12	0.74 ± 0.11	0.71 ± 0.14
	SET-PREDICT	0.64 ± 0.11	0.68 ± 0.09	0.63 ± 0.11
HEALTHY	SET-OBSERVE	0.78 ± 0.08	0.78 ± 0.07	0.70 ± 0.11
	SET-PREDICT	0.72 ± 0.09	0.68 ± 0.08	0.62 ± 0.10
PATIENTS	SET-OBSERVE	0.68 ± 0.18	0.74 ± 0.17	0.67 ± 0.16
	SET-PREDICT	0.58 ± 0.15	0.66 ± 0.18	0.57 ± 0.16

**Table 6:** Mean and standard deviation of balanced accuracy in test data across all subjects in the corresponding datasets for the 2-class classification approach.

Dataset	Feature Set	SVM	RF	LDA
ALL	SET-OBSERVE	0.87 ± 0.09	0.87 ± 0.08	0.86 ± 0.07
	SET-PREDICT	0.77 ± 0.14	0.78 ± 0.1	0.77 ± 0.13
HEALTHY	SET-OBSERVE	0.89 ± 0.07	0.91 ± 0.05	0.88 ± 0.05
	SET-PREDICT	0.84 ± 0.06	0.86 ± 0.05	0.81 ± 0.08
PATIENTS	SET-OBSERVE	0.82 ± 0.12	0.89 ± 0.06	0.77 ± 0.10
	SET-PREDICT	0.69 ± 0.16	0.76 ± 0.09	0.65 ± 0.15

## Data Availability

The data presented in this study are available upon reasonable request from the corresponding author.

## Abbreviations

The following abbreviations are used in this manuscript:

BMI	Body Mass Index
DIFF	Per value difference between double- and single-pulse MMG responses
EMG	Electromyography
IMU	Inertial Measurement Unit
LDA	Linear Discriminant Analysis
LOSO	Leave-One-Subject-Out
ML	Machine Learning
MMG	Mechanomyography
MS	Multiple Sclerosis
Q	Quadriceps
RF	Random Forest Classifier
SVM	Support Vector Machine
TS	Triceps Surae
tSCS	Transcutaneous Spinal Cord Stimulation
winA1	Window for feature extraction in single-pulse MMG signal 10–60 ms after first stimulus
winDIFF	Window for feature in DIFF extraction 10–60 ms after second stimulus
